# Crystal structure of *catena*-poly[[[aqua­bis­(1*H*-imidazole-κ*N* ^3^)copper(II)]-μ-3-({4-[(2-carboxyl­atoeth­yl)carbamo­yl]phen­yl}formamido)­propano­ato-κ^2^ *O*:*O*′] dihydrate]

**DOI:** 10.1107/S205698901500626X

**Published:** 2015-04-11

**Authors:** Yan Liu, Liu-Yang Xu, Hong-Tao Zhang

**Affiliations:** a College of Chemistry and Materials Science, Anhui Key Laboratory of Functional Molecular Solids, Anhui Normal University, Wuhu 241000, People’s Republic of China

**Keywords:** crystal structure, Cu^II^ coordination polymer, hydrogen bonding, C—H⋯π inter­actions

## Abstract

In the title polymeric complex, {[Cu(C_14_H_14_N_2_O_6_)(C_3_H_4_N_2_)_2_(H_2_O)]·2H_2_O}_*n*_, the Cu^II^ cation, located on a twofold rotation axis, is coordinated by one water mol­ecule and two imidazole mol­ecules as well as two symmetry-related 3-([4-[(2-carboxyl­atoeth­yl)carbamo­yl]phen­yl]formamido)­propano­ate dianions (*L* ^2−^) in an approximately square-pyramidal geometry. The coordinating water mol­ecule is located on a twofold rotation axis while the *L* ^2−^ anion sits about an inversion center. The *L* ^2−^ anions bridge the Cu^II^ cations, forming polymeric chains propagating along the [101] direction. In the crystal, O—H⋯O, N—H⋯O hydrogen bonds and weak C—H⋯π inter­action link the polymeric chains and the solvent water mol­ecules into a three-dimensional supra­molecular architecture.

## Related literature   

For related coordination polymers, see: Morrison *et al.* (2011 ▸); Wang *et al.* (2012 ▸); Zhang & Xiong (2012 ▸). For the synthesis, see: Yuan *et al.* (2002 ▸).
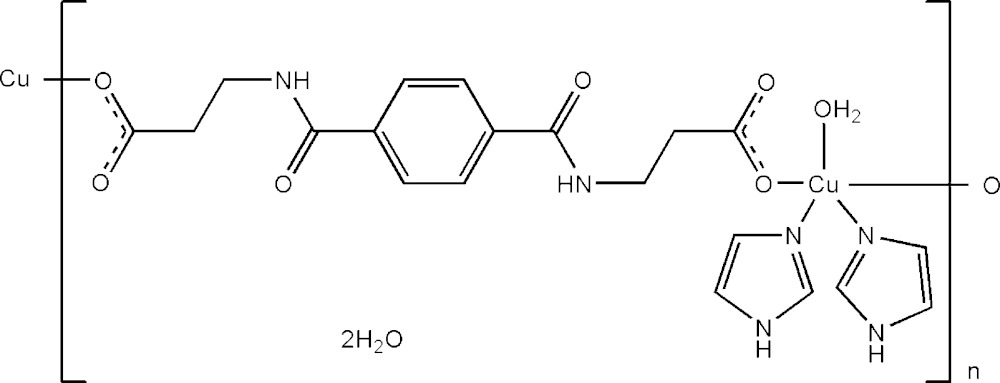


## Experimental   

### Crystal data  

[Cu(C_14_H_14_N_2_O_6_)(C_3_H_4_N_2_)_2_(H_2_O)]·2H_2_O
*M* *_r_* = 560.02
Monoclinic, 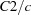

*a* = 27.752 (5) Å
*b* = 5.5793 (9) Å
*c* = 17.302 (3) Å
β = 115.855 (2)°
*V* = 2410.8 (7) Å^3^
*Z* = 4
Mo *K*α radiation
μ = 0.97 mm^−1^
*T* = 298 K
0.08 × 0.07 × 0.05 mm

### Data collection  

Bruker SMART APEXII CCD area-detector diffractometer
Absorption correction: multi-scan (*SADABS*; Bruker, 2004 ▸) *T*_min_ = 0.927, *T*_max_ = 0.958
9928 measured reflections
2775 independent reflections
1632 reflections with *I* > 2σ(*I*)
*R*_int_ = 0.081

### Refinement  

*R*[*F* ^2^ > 2σ(*F* ^2^)] = 0.053
*wR*(*F* ^2^) = 0.139
*S* = 0.99
2775 reflections
165 parameters
H-atom parameters constrained
Δρ_max_ = 0.58 e Å^−3^
Δρ_min_ = −0.36 e Å^−3^

### 

Data collection: *APEX2* (Bruker, 2004 ▸); cell refinement: *SAINT* (Bruker, 2004 ▸); data reduction: *SAINT*; program(s) used to solve structure: *SHELXTL* (Sheldrick, 2008 ▸); program(s) used to refine structure: *SHELXTL*; molecular graphics: *DIAMOND* (Brandenburg, 2008 ▸); software used to prepare material for publication: *SHELXTL*.

## Supplementary Material

Crystal structure: contains datablock(s) global, I. DOI: 10.1107/S205698901500626X/xu5844sup1.cif

Structure factors: contains datablock(s) I. DOI: 10.1107/S205698901500626X/xu5844Isup2.hkl

**Fig. 1.**
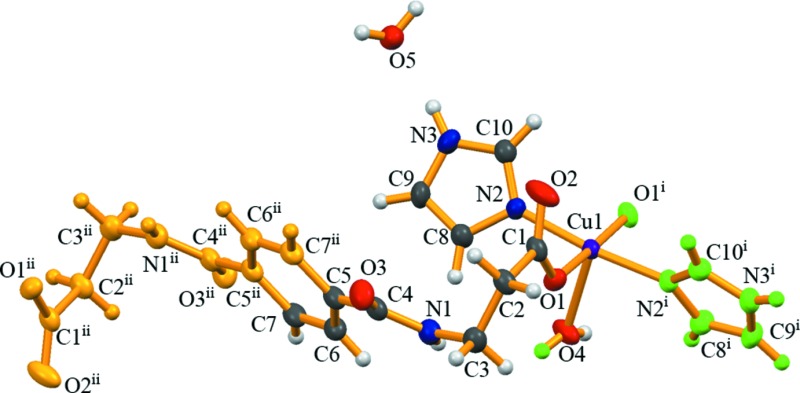
The molecular structure of (I), a drawing of the asymmetric unit (multi-colored portion) with displacement ellipsoids at the 30% probability level. [symmetry code: (i) 1 - x, y, -z + 3/2; (ii) -x + 1/2, -y + 3/2, -z + 1]

Click here for additional data file.x y z x y z . DOI: 10.1107/S205698901500626X/xu5844fig1.tif
The mol­ecular structure of (I), a drawing of the asymmetric unit (multi-colored portion) with displacement ellipsoids at the 30% probability level. [symmetry code: (i) 1 − *x*, *y*, −*z* + 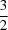; (ii) −*x* + 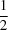, −*y* + 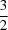, −*z* + 1]

**Fig. 2.**
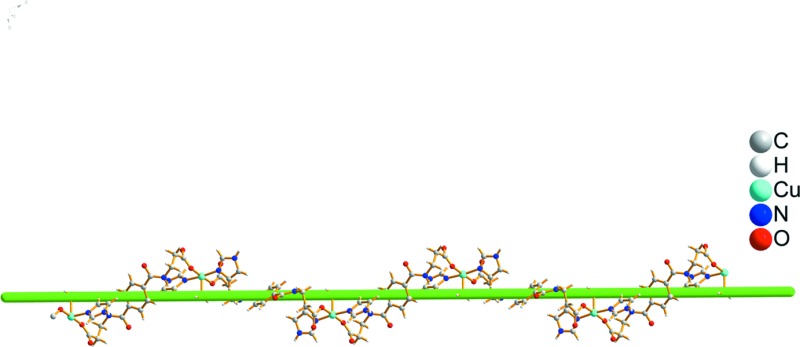
The polymeric chain of (I).

. DOI: 10.1107/S205698901500626X/xu5844fig2.tif
The polymeric chain of (I).

CCDC reference: 1056415

Additional supporting information:  crystallographic information; 3D view; checkCIF report

## Figures and Tables

**Table 1:** Selected bond lengths ()

Cu1N2	1.965(3)
Cu1O1	1.976(3)
Cu1O4	2.232(4)

**Table 2:** Hydrogen-bond geometry (, ) *Cg*1 is the centroid of the N2/N3/C8C10 imidazole ring.

*D*H*A*	*D*H	H*A*	*D* *A*	*D*H*A*
N1H1O2^i^	0.86	2.17	2.965(5)	153
N3H3O5	0.86	1.90	2.751(5)	172
O4H4O2^ii^	0.82	1.89	2.695(4)	167
O5H5*A*O3^iii^	0.85	1.91	2.731(4)	163
O5H5*B*O3^iv^	0.85	2.04	2.810(4)	149
C3H3*B* *Cg*1^v^	0.93	2.75	3.692(5)	164

Symmetry codes: (i) 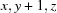; (ii) 
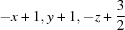
; (iii) 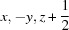; (iv) 
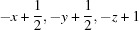
; (v) 
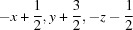
.

## supplementary crystallographic information

### S1. Comment

The self-assembly of coordination polymers have been one of the popular areas of in chemistry recently, owing to their intriguing structures and various physical properties, such as optical, electronic, magnetic and catalytic properties (Zhang *et al.* 2012, Wang *et al.* 2012). From the view point of the intermolecular interactions, the structures of the coordination polymers are mainly governed by the intra- and intermolecular interactions, such as the coordination interaction, hydrogen bonding interaction and π···π stacking as well as the molecular conformations depending on the molecular flexibility (Morrison *et al.* 2011). Accordingly, the aromatic pseudopeptidic molecules bearing the carboxylate groups could be the ideal building blocks to construct the coordination polymers with the metal ions. The σ-rotation about the N—C and the C—C bonds could induce flexibility in the molecules. The imine group N—H could serve as a better hydrogen-bonding donor and the amide C=O could also act as a better hydrogen-bonding acceptor. Moreover, the aromatic rings in the molecules could contribute to π···π stacking. The carboxlate groups in the molecules could capture the metal ions through the coordination interaction. Therefore, we have designed and synthesized an aromatic pseudopeptidic ligand, 3,3'-[1,4-bis(- benzamido)]dipropanoic acid (H_2_L). Here, we report the structure of the title helical chain-like coordination polymer, (I), which is derived from aromatic pseudopeptidic ligand, *L*^2-^, and imidazole, namely {[CuL(im)_2_(H_2_O)]2(H_2_O)}_n_ (im = imidazole), (I).

X-ray crystallographic analysis revealed that (I) crystallizes in the monoclinic space group *C*2/*c* with an asymmetric unit consisting of a divalent Cu^II^ cation and a coordinated water molecule residing on a twofold axis, half of an *L*^2-^ siting across a center of inversion and one imidazole as well as a lattice water molecule. As shown in Fig 1, the five-coordinate Cu^II^ cation has an approximately square-pyramidal coordination environment,in which the equatorial plane is composed of two N atoms (N2 and N2^i^, symmetry code:(i) 1 - *x*, *y*, -*z* + 3/2) from two imidazole molecules and two O atoms (O1 and O1^i^,symmetry code:(i) 1 - *x*, *y*, -*z* + 3/2) from two symmetry related *L*^2-^ dianions, while the water O atom (O4) occupies the apical position (Table 1). The apical Cu1—O4 distance is longer than the others,including the Cu—O1 and the Cu—N2 disatnces. The angles O1—Cu1—O1^i^ and N2—Cu—N2^i^ (symmetry code:(i) 1 - *x*, *y*, -*z* + 3/2) are of 177.05 (18) ° and 173.3 (2) °, respectively. It indicates that these five atoms are approximately located in an equatorial plane. The angles O4—Cu1—O1 and O4—Cu1—N2 are of 91.47 (9)° and 93.36 (10)°, which confirm an approximately square-pyramidal coordination geometry. The carboxylate group O1/C1/O2 coordinates to Cu^II^ cation in monodentate fashion *via* the atom O1. As a result, the ligand dianions connect the copper cations to construct an helical chain which runs along the [101] direction. In the formation of the single-strand helical structure, the peptide segment could play an important role. The torsion angle C1/C2/C3/N1 is of 59.8 (5)°, which is very close to 60 °. It shows the *gauche* conformation of the peptide segment. It also makes the ligand molecule twist twice and finally construct a single-strand helix through connecting the copper cations. It implies that the conformation of the ligand could be crucial in the formation of the helix. In the lattice, there are two types of helix, *viz*. right-handed and left-handed. Helices of the same type arrange in parallel along the *b* axis. The inter-chain hydrogen bonding interactions could contribute to the arrangement of the same type helices. The hydrogen bonding interactions between the lattice water molecules and the helices could contribute to the arrangement of the different type helices. However, the shortest center-center distance between two adjacent phenyl rings is beyond 5 Å. It indicates the absence of π···π staking in the crystal. Therefore, the helical chains are stabilized in the lattice mainly by the hydrogen bonding interactions and van der Waals interactions.

### S2. Experimental

The ligand H_2_L was synthesized from terephthaloyl chloride and β-alanine according to a similar method reported by Yuan *et al.* (2002). 13.6 mg (0.2 mmol) imidazole was dissolved in 10 ml water and then the ligand (30.8 mg, 0.1 mmol) was added in the solution. After H_2_L dissolved completely, The cupric acetate monohydrate (20.0 mg 0.1 mmol) was added in the solution. The resulting blue solution was filtered and the filtrate was left at room temperature. Blue block crystals of (I) were obtained (36.4 mg, yield *ca* 65%) after several weeks by slow evaporation of the solvent.

### S3. Refinement

H atoms were placed in calculated positions with C—H = 0.93–0.97 Å, O—H = 0.82–0.85 Å and N—H = 0.86 Å, and refined in riding mode.

### Crystal data

**Table:**

[Cu(C_14_H_14_N_2_O_6_)(C_3_H_4_N_2_)_2_(H_2_O)]·2H_2_O	*F*(000) = 1164
*M**_r_* = 560.02	*D*_x_ = 1.543 Mg m^−^^3^
Monoclinic, *C*2/*c*	Mo *K*α radiation, λ = 0.71073 Å
Hall symbol: -C 2yc	Cell parameters from 1477 reflections
*a* = 27.752 (5) Å	θ = 2.4–20.7°
*b* = 5.5793 (9) Å	µ = 0.97 mm^−^^1^
*c* = 17.302 (3) Å	*T* = 298 K
β = 115.855 (2)°	Block, blue
*V* = 2410.8 (7) Å^3^	0.08 × 0.07 × 0.05 mm
*Z* = 4	

### Data collection

**Table:**

Bruker SMART APEXII CCD area-detector diffractometer	2775 independent reflections
Radiation source: fine-focus sealed tube	1632 reflections with *I* > 2σ(*I*)
Graphite monochromator	*R*_int_ = 0.081
phi and ω scans	θ_max_ = 27.5°, θ_min_ = 1.6°
Absorption correction: multi-scan (*SADABS*; Bruker, 2004)	*h* = −35→36
*T*_min_ = 0.927, *T*_max_ = 0.958	*k* = −7→7
9928 measured reflections	*l* = −22→22

### Refinement

**Table:**

Refinement on *F*^2^	0 restraints
Least-squares matrix: full	H-atom parameters constrained
*R*[*F*^2^ > 2σ(*F*^2^)] = 0.053	*w* = 1/[σ^2^(*F*_o_^2^) + (0.0621*P*)^2^] where *P* = (*F*_o_^2^ + 2*F*_c_^2^)/3
*wR*(*F*^2^) = 0.139	(Δ/σ)_max_ < 0.001
*S* = 0.99	Δρ_max_ = 0.58 e Å^−^^3^
2775 reflections	Δρ_min_ = −0.36 e Å^−^^3^
165 parameters	

### Special details

**Table:**

Geometry. All e.s.d.'s (except the e.s.d. in the dihedral angle between two l.s. planes) are estimated using the full covariance matrix. The cell e.s.d.'s are taken into account individually in the estimation of e.s.d.'s in distances, angles and torsion angles; correlations between e.s.d.'s in cell parameters are only used when they are defined by crystal symmetry. An approximate (isotropic) treatment of cell e.s.d.'s is used for estimating e.s.d.'s involving l.s. planes.
Refinement. Refinement of *F*^2^ against ALL reflections. The weighted *R*-factor *wR* and goodness of fit *S* are based on *F*^2^, conventional *R*-factors *R* are based on *F*, with *F* set to zero for negative *F*^2^. The threshold expression of *F*^2^ > σ(*F*^2^) is used only for calculating *R*-factors(gt) *etc*. and is not relevant to the choice of reflections for refinement. *R*-factors based on *F*^2^ are statistically about twice as large as those based on *F*, and *R*- factors based on ALL data will be even larger.

### Fractional atomic coordinates and isotropic or equivalent isotropic displacement parameters (Å^2^)

**Table:**

	*x*	*y*	*z*	*U*_iso_*/*U*_eq_	
C1	0.44842 (17)	0.1862 (8)	0.5751 (3)	0.0456 (11)	
C2	0.42650 (18)	0.2325 (8)	0.4802 (2)	0.0498 (11)	
H2A	0.3936	0.1421	0.4512	0.060*	
H2B	0.4519	0.1722	0.4603	0.060*	
C3	0.41500 (19)	0.4960 (9)	0.4541 (3)	0.0560 (12)	
H3A	0.4483	0.5854	0.4803	0.067*	
H3B	0.4018	0.5081	0.3923	0.067*	
C4	0.32380 (18)	0.5588 (8)	0.4379 (3)	0.0494 (11)	
C5	0.28700 (17)	0.6644 (8)	0.4710 (3)	0.0473 (11)	
C6	0.29707 (18)	0.8736 (8)	0.5187 (3)	0.0516 (11)	
H6	0.3285	0.9585	0.5317	0.062*	
C7	0.26074 (18)	0.9564 (8)	0.5468 (3)	0.0520 (12)	
H7	0.2682	1.0972	0.5787	0.062*	
C8	0.38648 (18)	0.5107 (8)	0.6976 (3)	0.0516 (11)	
H8	0.3900	0.6493	0.6707	0.062*	
C9	0.34147 (18)	0.4422 (9)	0.7033 (3)	0.0553 (12)	
H9	0.3090	0.5235	0.6825	0.066*	
C10	0.40408 (18)	0.1793 (8)	0.7653 (3)	0.0494 (11)	
H10	0.4216	0.0426	0.7951	0.059*	
Cu1	0.5000	0.36573 (12)	0.7500	0.0343 (2)	
N1	0.37603 (14)	0.6062 (6)	0.4788 (2)	0.0509 (9)	
H1	0.3872	0.7051	0.5211	0.061*	
N2	0.42632 (12)	0.3451 (6)	0.73776 (19)	0.0404 (8)	
N3	0.35351 (14)	0.2310 (7)	0.7454 (2)	0.0526 (10)	
H3	0.3320	0.1446	0.7573	0.063*	
O1	0.47315 (11)	0.3567 (5)	0.62383 (16)	0.0461 (7)	
O2	0.43997 (16)	−0.0138 (6)	0.59984 (19)	0.0772 (11)	
O3	0.30600 (12)	0.4261 (6)	0.3745 (2)	0.0640 (9)	
O4	0.5000	0.7658 (7)	0.7500	0.0613 (13)	
H4	0.5172	0.8147	0.7992	0.092*	
O5	0.28059 (12)	−0.0023 (6)	0.7868 (2)	0.0658 (9)	
H5A	0.2944	−0.1316	0.8131	0.079*	
H5B	0.2528	−0.0347	0.7410	0.079*	

### Atomic displacement parameters (Å^2^)

**Table:**

	*U*^11^	*U*^22^	*U*^33^	*U*^12^	*U*^13^	*U*^23^
C1	0.047 (3)	0.047 (3)	0.033 (2)	0.009 (2)	0.009 (2)	−0.002 (2)
C2	0.057 (3)	0.049 (3)	0.034 (2)	0.007 (2)	0.011 (2)	−0.002 (2)
C3	0.053 (3)	0.068 (3)	0.043 (3)	0.002 (2)	0.017 (2)	0.006 (2)
C4	0.050 (3)	0.047 (3)	0.038 (2)	0.004 (2)	0.006 (2)	0.005 (2)
C5	0.051 (3)	0.039 (3)	0.037 (2)	0.002 (2)	0.006 (2)	−0.0019 (19)
C6	0.046 (3)	0.045 (3)	0.049 (3)	−0.005 (2)	0.007 (2)	−0.002 (2)
C7	0.055 (3)	0.039 (3)	0.046 (3)	−0.002 (2)	0.008 (2)	−0.006 (2)
C8	0.049 (3)	0.057 (3)	0.048 (3)	0.007 (2)	0.020 (2)	0.009 (2)
C9	0.036 (3)	0.068 (3)	0.056 (3)	0.007 (2)	0.015 (2)	0.005 (3)
C10	0.047 (3)	0.050 (3)	0.048 (3)	0.003 (2)	0.018 (2)	0.000 (2)
Cu1	0.0316 (4)	0.0344 (4)	0.0312 (4)	0.000	0.0084 (3)	0.000
N1	0.049 (2)	0.052 (2)	0.042 (2)	−0.0002 (19)	0.0110 (18)	−0.0060 (17)
N2	0.0350 (19)	0.047 (2)	0.0358 (18)	−0.0022 (17)	0.0122 (15)	0.0007 (16)
N3	0.041 (2)	0.063 (3)	0.054 (2)	−0.0093 (19)	0.0211 (19)	−0.006 (2)
O1	0.0432 (17)	0.0548 (18)	0.0342 (15)	−0.0116 (15)	0.0112 (13)	−0.0034 (14)
O2	0.116 (3)	0.040 (2)	0.0441 (19)	0.000 (2)	0.0056 (19)	0.0014 (16)
O3	0.058 (2)	0.061 (2)	0.054 (2)	−0.0011 (17)	0.0068 (17)	−0.0206 (16)
O4	0.075 (3)	0.038 (2)	0.040 (2)	0.000	−0.004 (2)	0.000
O5	0.052 (2)	0.063 (2)	0.069 (2)	−0.0075 (17)	0.0134 (17)	0.0111 (18)

### Geometric parameters (Å, º)

**Table:**

C1—O2	1.253 (5)	C8—C9	1.350 (6)
C1—O1	1.259 (5)	C8—N2	1.374 (5)
C1—C2	1.503 (5)	C8—H8	0.9300
C2—C3	1.531 (6)	C9—N3	1.348 (6)
C2—H2A	0.9700	C9—H9	0.9300
C2—H2B	0.9700	C10—N2	1.312 (5)
C3—N1	1.460 (5)	C10—N3	1.323 (5)
C3—H3A	0.9700	C10—H10	0.9300
C3—H3B	0.9700	Cu1—N2^ii^	1.965 (3)
C4—O3	1.234 (5)	Cu1—N2	1.965 (3)
C4—N1	1.333 (5)	Cu1—O1^ii^	1.976 (3)
C4—C5	1.492 (6)	Cu1—O1	1.976 (3)
C5—C6	1.386 (6)	Cu1—O4	2.232 (4)
C5—C7^i^	1.396 (6)	N1—H1	0.8600
C6—C7	1.377 (6)	N3—H3	0.8600
C6—H6	0.9300	O4—H4	0.8200
C7—C5^i^	1.396 (6)	O5—H5A	0.8500
C7—H7	0.9300	O5—H5B	0.8500
			
O2—C1—O1	124.9 (4)	N2—C8—H8	125.1
O2—C1—C2	118.7 (4)	N3—C9—C8	105.5 (4)
O1—C1—C2	116.4 (4)	N3—C9—H9	127.3
C1—C2—C3	114.9 (4)	C8—C9—H9	127.3
C1—C2—H2A	108.5	N2—C10—N3	111.4 (4)
C3—C2—H2A	108.5	N2—C10—H10	124.3
C1—C2—H2B	108.5	N3—C10—H10	124.3
C3—C2—H2B	108.5	N2^ii^—Cu1—N2	173.3 (2)
H2A—C2—H2B	107.5	N2^ii^—Cu1—O1^ii^	90.35 (12)
N1—C3—C2	113.8 (4)	N2—Cu1—O1^ii^	89.48 (12)
N1—C3—H3A	108.8	N2^ii^—Cu1—O1	89.48 (12)
C2—C3—H3A	108.8	N2—Cu1—O1	90.35 (11)
N1—C3—H3B	108.8	O1^ii^—Cu1—O1	177.09 (18)
C2—C3—H3B	108.8	N2^ii^—Cu1—O4	93.36 (10)
H3A—C3—H3B	107.7	N2—Cu1—O4	93.36 (10)
O3—C4—N1	120.7 (4)	O1^ii^—Cu1—O4	91.46 (9)
O3—C4—C5	120.1 (4)	O1—Cu1—O4	91.46 (9)
N1—C4—C5	119.1 (4)	C4—N1—C3	122.3 (4)
C6—C5—C7^i^	117.6 (4)	C4—N1—H1	118.8
C6—C5—C4	124.5 (4)	C3—N1—H1	118.8
C7^i^—C5—C4	117.9 (4)	C10—N2—C8	104.8 (4)
C7—C6—C5	120.3 (4)	C10—N2—Cu1	129.4 (3)
C7—C6—H6	119.8	C8—N2—Cu1	125.8 (3)
C5—C6—H6	119.8	C10—N3—C9	108.5 (4)
C6—C7—C5^i^	122.1 (4)	C10—N3—H3	125.8
C6—C7—H7	119.0	C9—N3—H3	125.8
C5^i^—C7—H7	119.0	C1—O1—Cu1	126.4 (3)
C9—C8—N2	109.8 (4)	Cu1—O4—H4	109.5
C9—C8—H8	125.1	H5A—O5—H5B	109.5

Symmetry codes: (i) −*x*+1/2, −*y*+3/2, −*z*+1; (ii) −*x*+1, *y*, −*z*+3/2.

### Hydrogen-bond geometry (Å, º)

Cg1 is the centroid of the N2/N3/C8–C10 imidazole ring.

**Table:**

*D*—H···*A*	*D*—H	H···*A*	*D*···*A*	*D*—H···*A*
N1—H1···O2^iii^	0.86	2.17	2.965 (5)	153
N3—H3···O5	0.86	1.90	2.751 (5)	172
O4—H4···O2^iv^	0.82	1.89	2.695 (4)	167
O5—H5*A*···O3^v^	0.85	1.91	2.731 (4)	163
O5—H5*B*···O3^vi^	0.85	2.04	2.810 (4)	149
C3—H3*B*···*Cg*1^vii^	0.93	2.75	3.692 (5)	164

Symmetry codes: (iii) *x*, *y*+1, *z*; (iv) −*x*+1, *y*+1, −*z*+3/2; (v) *x*, −*y*, *z*+1/2; (vi) −*x*+1/2, −*y*+1/2, −*z*+1; (vii) −*x*+1/2, *y*+3/2, −*z*−1/2.
