# Supplementary figures and images for: Crystal structure of catena-poly[[[aqua­bis­(1H-imidazole-κN 3)copper(II)]-μ-3-({4-[(2-carboxyl­atoeth­yl)carbamo­yl]phen­yl}formamido)­propano­ato-κ2 O:O′] dihydrate]
Source: Acta Crystallogr E Crystallogr Commun. 2015 Apr 11;71(Pt 5):m108–9. doi: 10.1107/S205698901500626X (PMC4420079; doi:10.1107/S205698901500626X)

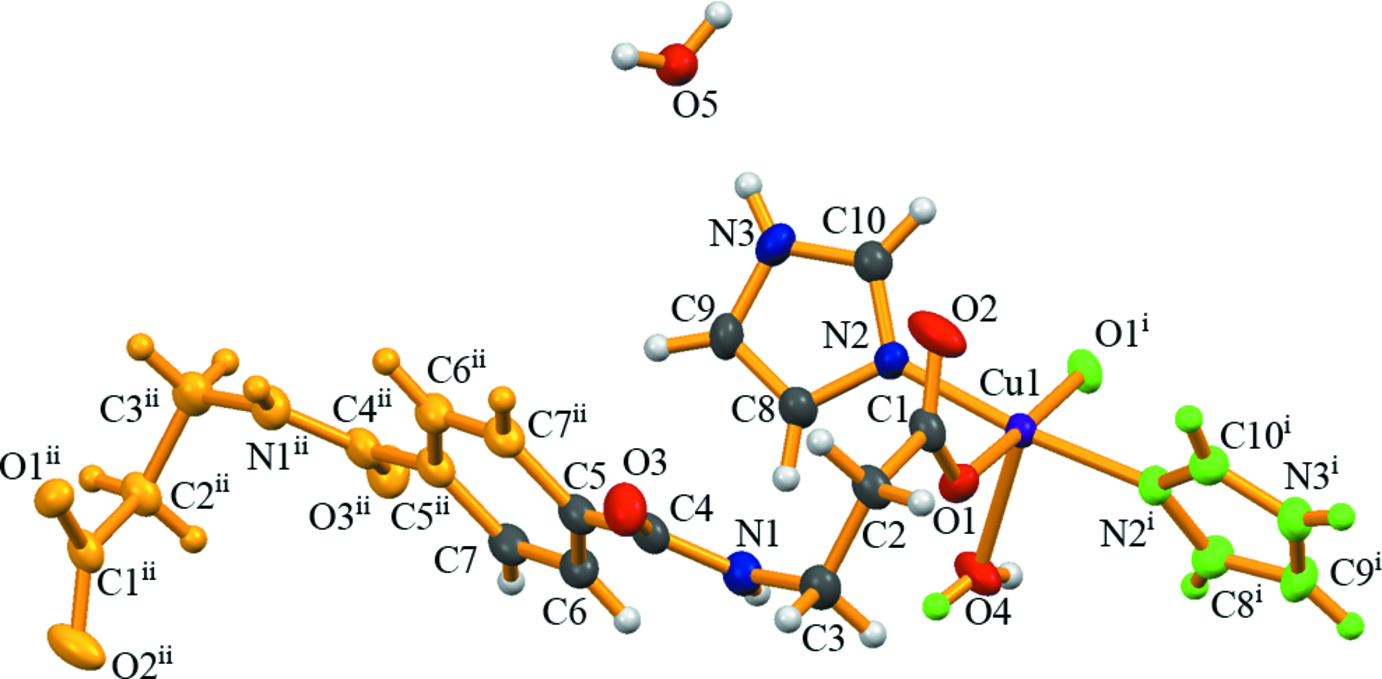

Supplement: Supplementary file 3 [file e-71-0m108-fig1.tif]

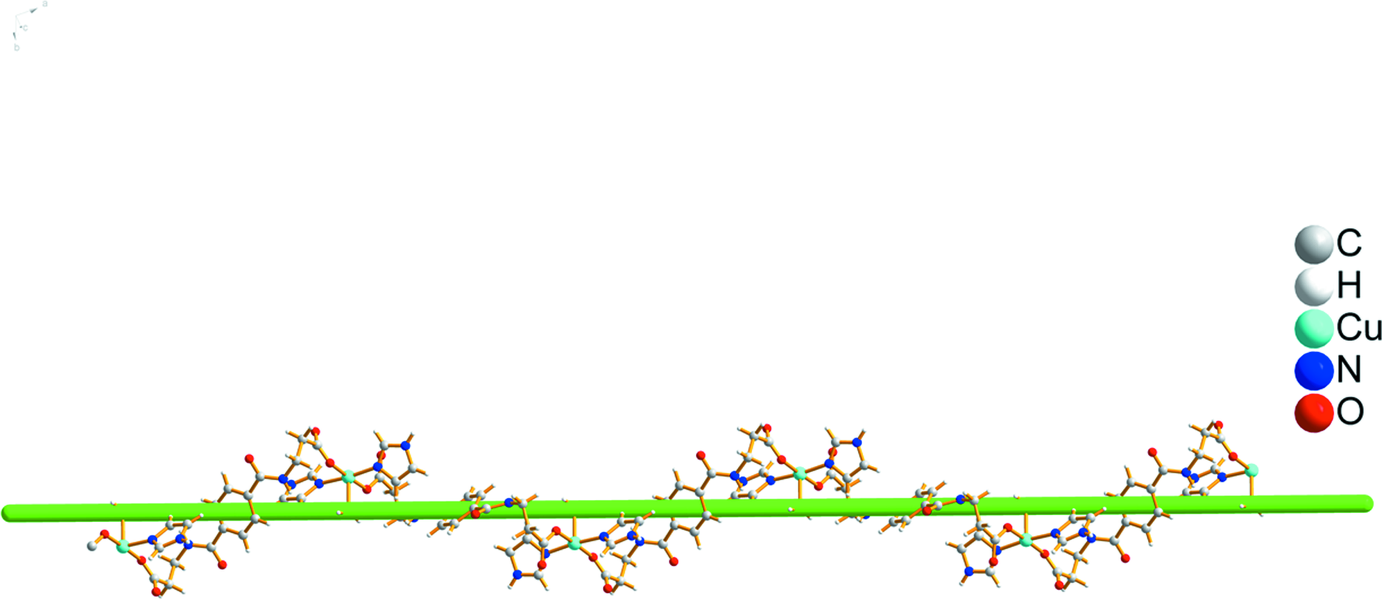

Supplement: Supplementary file 4 [file e-71-0m108-fig2.tif]
